# In silico analysis for SARS-CoV-2 detection in the context of genetic variability of the Algerian omicron variant 

**DOI:** 10.22099/mbrc.2024.50192.1985

**Published:** 2024

**Authors:** Chahinez Amira Dahmani, Asmaa Azzoune, Abdallah Boudjema

**Affiliations:** 1 Biology Department, Faculty of Natural and Life Sciences, University of Mostaganem, Algeria; 2 Laboratory of Molecular and Cellular Genetics (LGMC), University of Sciences and Technology of Oran, Algeria; 3 High School of Biological Sciences, Oran, Algeria

**Keywords:** AlgeriaAlignmentoOmicron variant

## Abstract

The risk to public health conferred by the Omicron variant is still not completely clear, although its numerous gene mutations have raised concerns regarding its potential for increased transmissibility and immune escape. In this study, we test the compatibility of the different primers and probes available in different commercial kits sold internationally with all the sequences of SARS-CoV-2 analyzed in Algeria until March 2023. The Algerian SARS-CoV-2 Omicron variant sequences were aligned with the Muscle tool using Genious software. We also used primers and probes sequences of seven international RT-qPCR kits; CDC China, Charite Germany, HKU Hong Kong, NIH Thailand, NIID Japan, CDC US, and Pasteur Institute. We used the primer check v2.0 developed by VIROSCIENCE LAB, To identify the different mutations located at the level of primers and probes about the Algerian sequences of SARS-CoV2. Statistical tests were carried out by calculating the $x^{2}$ test. We found regarding the Forward primer sequences that the two Thailand and Japan kits are less specific to the Algerian version of the SARS-CoV-2 Omicron variant genome compared to the other kits (*p*=10^-6^). Furthermore, regarding the Reverse primers and fluorescent Probes, the three kits; Thailand, Japan, and CDC US; are less effective (*p*=10^-6^). Regarding all primers and probes, this work allowed us to conclude that the four RT-qPCR kits: CDC China, Charite Germany, NHD Hong Kong, and Pasteur Institute seem to be more specific for the Algerian omicron genome detection and therefore for diagnosis of COVID-19 in Algeria.

## INTRODUCTION

The International Committee for the Taxonomy of Viruses (ICTV) endorsed the following definition for a virus species in 1991: “A virus species is a polythetic class of viruses that constitute a replicating lineage and occupy a particular ecological niche”. For Coronavirus, the ICTV coronavirus study group has suggested a species criterion based on rooted phylogenies and pairwise amino acid distances in seven concatenated domains of the non-structural part of the COVID genome [1,2].

At the beginning of 2020, it became clear that a new pathogenic human coronavirus, named novel coronavirus (2019-nCoV), had appeared in Wuhan, China [3]. On March 11, 2020, the World Health Organization (WHO) declared a global pandemic caused by the novel coronavirus, SARS-Cov-2, the causative agent of a respiratory disease known as the infectious disease due to the new coronavirus (COVID-19; Coronavirus Disease 2019). Since then, severe Acute Respiratory Syndrome Coronavirus 2 (SARS-COV-2) has evolved rapidly, and on November 26, 2021, WHO designated Omicron (lineage B.1.1.529) SARS-CoV-2, in the first known case of this variant that was detected in South Africa [4].

The first case of COVID-19 in Algeria was reported on February 25, 2020, in the city of Ouargla, this case was imported from Italy. Soon after, several hundred or even thousands of cases of COVID-19 were diagnosed in the country. Until May 25, 2022, it reached 42,619 confirmed cases, it is the most affected country in Africa and the third country in terms of deaths with 1465 deaths only in 2020 [5].

From the start of the COVID-19 epidemic, bioinformatics has shown its usefulness. After the detection in December 2019 of the first patients infected with the virus, Chinese scientists embarked on the sequencing of the viral genome of this new pathogen from samples taken from patients. Composed of some 30,000 nucleotides, the SARS-CoV-2 genome is then revealed in record time and all revealed sequences of this viral RNA are available on the GISAID database (www.gisaid.org (http://www.gisaid.org/)). At the end of June 2020, the GISAID EpiCoV database held more than 57,000 genome sequences of SARS-CoV-2.

Coronaviruses (CoV) (order *Nidovirales*, family *Coronaviridae*, subfamily *Coronavirinae*) are positive-strand RNA viruses. The subfamily *Coronavirinae* contains the four genera *Alpha-, Beta-, Gamma*-, and *Deltacoronavirus* [2]. The SARS-CoV-2 genome is composed of a single-stranded positive-sense RNA and measures approximately 29.9 kb including 25 genes. SARS-CoV-2 has a variable number of small ORFs present between the different conserved genes (ORF1ab), Spike (S), Envelope (E), Membrane (M), and Nucleocapsid (N). The viral genome contains distinctive features including the unique N-terminal fragment for the major structural proteins of all coronaviruses that occur in the 5'-3' order as S, E, M, and N. There are also several non-structural proteins, such as NSP1 to NSP10 and NSP12 to NSP16, encoded by genes located in the 5' region of the viral RNA genome [6, 7].

Since the start of the COVID-19 pandemic, various methods for the diagnosis of SARS-CoV-2 have been reported in the literature, including the reference molecular biology method; real-time RT-PCR (RT-qPCR). At present, in the context of large-scale screening, RT-qPCR testing remains the standard for the diagnosis of COVID-19 despite the false-negative rate [8]. Several commercial kits have been designed to detect the viral genome of SARS-CoV-2. Each of the kits represented a set of specific primers or probe/specific primer pairs. However, several virus genomes with mutations in the primer and probe design regions have been detected in some publications [9-11]. These mutations may have no effect or turn into an opportunity for rapid molecular screening of variants. Therefore, data on the whole genome of SARS-CoV-2 strains in each geographical area contributes to the knowledge of its variability and the development of specific diagnostics.

Mutations of SARS-CoV-2 occur naturally during the phenomenon of replication. Thousands of mutations have accumulated and continue to occur. As new mutations continue to appear on the scene, new variants are increasingly being observed [12, 13]. The public health risk conferred by the Omicron variant is still not completely clear, although its numerous genetic mutations have suggested its great potential for variability and contagion. According to CovSPECTRUM (https://cov-spectrum.org/about**)**, 20,243 Omicron Variants were registered with the Global Initiative on Sharing All Influenza Data (GISAID) in December 2021 [10]. Recent results have demonstrated that the Omicron BA.1 lineage can deviate further from its (already mutated) genome and that patients with persistent infections can also transmit these viral variants. This study highlights an urgent need to implement strategies to prevent prolonged replication of SARS-CoV-2 and limit the spread of new emerging variants [14, 15].

In this work, we were interested in precisely this last variant which raised concerns about its potential to increase transmissibility and immune evasion. We carried out a bioinformatics study using data available on the GISAID database concerning the sequencing of SARS-CoV-2 in Algeria given the absence of specific or local diagnostic kits. Towards the end of November 2021, the team of *K.L. *Brown et al, 2022 found for the first time a single mismatch between the Omicron sequence and one of the primers in their assay which caused a delay>4 cycles during amplification [11]. The primer-template mismatch was then used as a quick surrogate marker for Omicron. The objective of our research was to test the compatibility of the different primers and/or probes selected in different commercial kits available internationally with the SARS-CoV-2 sequences analyzed in Algeria.

## MATERIALS AND METHODS

This study was designed following the ethical standards of the 1964 Declaration of Helsinki and its subsequent amendments. The information needed for this study was taken from previously anonymized data sources and does not pose a risk to the community.

**Analysis of publishing SARS-CoV2 sequences:** The SARS-CoV-2 Omicron genome sequences were extracted from the Global Initiative on Sharing Avian Influenza Data-EpiCoV (GISAID-EpiCoV) platform (https://gisaid.org/) [16], a global science initiative and a primary source established in 2008 that provides open access to genomic data of influenza viruses and the coronavirus responsible for the COVID-19 pandemic. Preference was given to complete sequences excluding low coverage sequences. Using the filter tool, all COVID-19 sequence data from Algeria were available but only the Omicron variant sequences were selected. All the sequence files were saved and downloaded in FASTA format, the gene length of which is 29,903 base pairs. These sequences were compared to the reference sequence (GENEBANK, National Center for Biotechnology Information NCBI, NC_045512-2).

**Analysis of primer and probe sequences: **The genetic sequences for primers and probes were used from seven international real-time RT-qPCR kits; CDC China, Charite Germany, HKU Hong Kong (China), NIH Thailand, NIID Japan, CDC US (USA), and Pasteur Institute (France). Each primer and probe sequences are detailed in Table 1.

**Alignment of SARS-COV-2 sequences: **This part consisted of aligning all the Algerian sequences of the Variant Omicron. The sequences were aligned with the Muscle tool [17], using the GENIOUS software. Missing sequences were excluded from the analysis.

**Identification of mutations in primer and probe sequences: **The identification of the different mutations located at the level of the primers and the probes of the seven kits was carried out in Tables 4, 5 and 6. For this, the latest primer check website was used: primer check v2.0 developed by *VIROSCIENCE LAB* and using the *GISAID* database. It is an excellent tool for bioinformatics because it allows testing of all available kits, in particular the seven that we used according to the different aligned sequences. After loading the sequences into Primer check 2.0, we tested the seven different real-time RT-qPCR kits for each sequence: CDC China, Charite Germany, NHD Hong Kong, NIID Japan, CDC US, and Pasteur Institute Assay (France). 

**Comparison of our study with reference data:** Finally, the European Centre for Disease Prevention and Control (ECDC-PRIMER SCAN WEBSITE) was used for the primers and probes mutations comparison of our study. The mutation frequencies collected in part of our study were compared with the trends observed in ECDC reports. Subsequently, similarities and differences between populations and regions were identified.

**Statistical Analysis: **The analysis of the distribution of the various mismatches between the various aligned SARS-CoV-2 sequences and the sequences of the primers and the probes was carried out by calculating the chi-square test (*p*<0.05) using the Statistical Package for Social Sciences (SPSS) software version 23.0 for Windows (SPSS Inc.). For the multiple combinations made in Tables 4 and 5, Bonferroni's corrections (*p*<10^-3^) were applied.

## RESULTS AND DISCUSSION

It has been reported that SARS-CoV-2 has experienced more than 10,000 mutations compared to the reference genome collected in January 2020 [18, 19]. In general, RNA viruses are prone to random mutations but nidoviruses, including coronaviruses, possess an enzyme to excise erroneous nucleotides and thus maintain good precision in virus replication and transcription [20]. The global and rapid emergence of COVID-19 has provided the virus with substantial opportunities for a natural selection of rare but favourable mutations. Although most viral mutations are benign, many mutations enhance viral survivability [21]. Thousands of mutations have persisted since the emergence of the virus. It turned out that the S-glycoprotein "RBD" (Receptor Binding Domain) is an essential determinant of the viral infectivity of SARS-COV-2, therefore only mutations located in this region will modify the affinity of the RBD with its ACE2 receptor [12]. Some authors have also shown that the presence of SARS-CoV-2 mutations can have a negative impact on the diagnostic test for COVID-19, or turn into an opportunity for molecular screening of variants [11]. 

All this information indicates that it is necessary to choose the viral nucleotide material well to establish a reliable diagnosis. As a result, almost all COVID-19 diagnostic kits commercialized on the international market have not taken the *S *gene sequence as a reference molecular marker. In Table 1, we can see that the seven kits used in this study rather selected the genes, N, E, RdRP, ORF1ab. The Japanese kit was the only one to have used oligonucleo-tides on the S genes but they were coupled with sets of primers and probes on the *N* and *ORF1a* genes (Table 1).

For the first part of this study, 446 Algerian SARS-CoV-2 sequences taken in January 2023 were collected concerning the Omicron variant. In the beginning, all the available Algerian sequences were selected concerning all the variants. However, due to the emergence of the Omicron variant during the year 2022, we decided to select only this last variant. Furthermore, all mismatches observed using the seven RT-qPCR kits after the alignment were presented in Table 2. Among the 446 Algerian sequences, we found a total of 461 mismatches between all primers and probes in the seven RT-qPCR kits. These mismatches were classified according to the seven RT-qPCR kits. Indeed, we observed a very high total mismatch rate for the *NIID Japan* and *CDC US* kits (149 vs. 134, respectively) and a rather average rate for the HKU kits. Hong Kong, CDC China, and NIH Thailand kits (63, 55, and 43, respectively). On the other hand, we observed for the two kits “Charite Germany and Pasteur Institute” a low total mismatch rate (08 vs. 09, respectively). This distribution showed a statistically significant difference (p= 0.04) between the different kits used for the diagnosis of COVID-19.

Then, this result motivated us to further stratify the different mismatches found according to their position either on the forward primers (F), the reverse primers (R), or the probes (P). As mentioned in Table 2, these mutations were classified according to the oligonucleotides used by each RT-qPCR kit. Indeed, we observed a significant distribution between each type of oligonucleotides used and the seven selected RT-qPCR kits (Forward = 0.01, Reverse= 0.02, and Probe= 0.02). Furthermore, we can see in Table 2 that the mutations in the fluorescent probes are less frequent (25%) compared to the primers (75%) in different COVID-19 diagnostic kits. 

**Table 1:** Primers and probes sequences of the seven RT-qPCR diagnostic kits for COVID-19 according to GISAID database (https://www.gisaid.org/ January 2023).

**RT-qPCR Kit**	**Targets**	**Oligonucleotides**	**Sequence **
**CDC China Kit**	**ORF1b gene** **N gene**	Target 1 (ORF1ab) F Target 1 (ORF1ab) R Target 1 (ORF1ab) P Target 2 (N) F Target 2 (N) R Target 2 (N) P	CCCTGTGGGTTTTACACTTAA ACGATTGTGCATCAGCTGA CCGTCTGCGGTATGTGGAAAGGTTATGG GGGGAACTTCTCCTGCTAGAAT CAGACATTTTGCTCTCAAGCTG TTGCTGCTGCTTGACAGATT
**Charite Germany kit**	**RdRP gene** **E gene**	RdRP_SARSr-F2 RdRP_SARSr-R1 RdRP_SARSr-P1 RdRP_SARSr-P2 E_Sarbeco_F1 E_Sarbeco_R2 E_Sarbeco_P1	GTGARATGGTCATGTGTGGCGG CARATGTTAAASACACTATTAGCATA CCAGGTGGWACRTCATCMGGTGATGC CAGGTGGAACCTCATCAGGAGATGC ACAGGTACGTTAATAGTTAATAGCGT ATATTGCAGCAGTACGCACACA ACACTAGCCATCCTTACTGCGCTTCG
**HKU. Hong Kong kit**	**ORF1b gene** **N gene**	HKU-ORF1b-nsp14F HKU-ORF1b nsp14R HKU-ORF1b nsp141P HKU-NF HKU-NR HKU-NP	TGGGGYTTTACRGGTAACCT AACRCGCTTAACAAAGCACTC TAGTTGTGATGCWATCATGACTAG TAATCAGACAAGGAACTGATTA CGAAGGTGTGACTTCCATG CCGCAAATTGCACAATTTGC
**NIH Thailand kit**	**N gene**	WH-NIC N-F WH-NIC N-R WH-NIC N-P	CGTTTGGTGGACCCTCAGAT CCCCACTGCGTTCTCCATT CAACTGGCAGTAACCA
	**N gene** **ORF1A gene**	NIID_2019nCOV_N_F2 NIID_2019-nCOV_N_R2 NIID_2019-nCOV_N_P2 NIID_WH-1_F501	AAATTTTGGGGACCAGGAAC TGGCAGCTGTGTAGGTCAAC ATGTCGCGCATTGGCATGGA TTCGGATGCTCGAACTGCACC
**NIID Japan kit**	**S gene**	NIID_WH-1_R913 NIID_WH-1_F509 NIID_WH-1_R854 NIID_WH-1_Seq_F519 NIID_WH-1_Seq_R840 WuhanCoV-spk1-f WuhanCoV-spk2-r NIID_WH-1_F24381 NIID_WH-1_R24873 NIID_WH-1_Seq_F24383 NIID_WH-1_Seq_R24865	CTTTACCAGCACGTGCTAGAAGG CTCGAACTGCACCTCATGG CAGAAGTTGTTATCGACATAGC ACCTCATGGTCATGTTATGG GACATAGCGAGTGTATGCC TTGGCAAAATTCAAGACTCACTTT TGTGGTTCATAAAAATTCCTTTGTG TCAAGACTCACTTTCTTCCAC ATTTGAAACAAAGACACCTTCAC AAGACTCACTTTCTTCCACAG CAAAGACACCTTCACGAGG
**CDC ** **US kit**	**N1 gene** **N2 gene** **N3 gene**	2019-nCoV_N1-F 2019-nCoV_N1-R 2019-nCoV_N1-P 2019-nCoV_N2-F 2019-nCoV_N2-R 2019-nCoV_N2-P 2019-nCoV_N3-F 2019-nCoV_N3-R 2019-nCoV_N3-P	GACCCCAAAATCAGCGAAAT TCTGGTTACTGCCAGTTGAATCTG ACCCCGCATTACGTTTGGTGGACC TTACAAACATTGGCCGCAAA GCGCGACATTCCGAAGAA ACAATTTGCCCCCAGCGCTTCAG GGGAGCCTTGAATACACCAAAA TGTAGCACGATTGCAGCATTG AYCACATTGGCACCCGCAATCCTG
**Pasteur Institute kit**	**RdRP gene** **RdRP gene** **E gene**	nCoV_IP2-12669Fw nCoV_IP2-12759Rv nCoV_IP2-12696bProbe nCoV_IP4-14059Fw nCoV_IP4-14146Rv nCoV_IP4-14084Probe E_Sarbeco_F1-Pasteur E_Sarbeco_R2-Pasteur E_Sarbeco_P1-Pasteur	ATGAGCTTAGTCCTGTTG CTCCCTTTGTTGTGTTGT AGATGTCTTGTGCTGCCGGTA GGTAACTGGTATGATTTCG CTGGTCAAGGTTAATATAGG TCATACAAACCACGCCAGG ACAGGTACGTTAATAGTTAATAGCGT ATATTGCAGCAGTACGCACACA ACACTAGCCATCCTTACTGCGCTTCG

**Table 2:** Comparison between the number of mismatches found in primers and probes of RT-qPCR kits according to aligned SARS-CoV-2 sequences.

**RT-qPCR kits**	**Total ** **of Mismatches** **n=461 (100%)**	**Reverse mismatches ** **n (%)**	**Probes mismatches ** **n (%)**	**Forward mismatches ** **n (%)**
**CDC China Kit**	55	23(42)	21(38)	11(20)
**Charite Germany kit**	08	01(12)	03(38)	04(50)
**HKU. Hong Kong kit**	63	20(32)	20(32)	23(36)
**NIH Thailand kit**	43	19 (44)	04 (10)	20 (46)
**NIID Japan kit**	149	68 (46)	20(13)	61(41)
**CDC US kit**	134	27 (20)	48 (36)	59 (44)
**Pasteur Institute kit**	09	06 (67)	00 (00)	03 (33)
**P-value**	0.04	0.02	0.02	0.01

Nevertheless, some kits show more mismatches on the Forward primer sequences like the Charite Germany and CDC US kits, and others on the Reverse primer sequences like the CDC China and Pasteur Institute kits. The others had rather similar frequencies (Table 2). 

One of the key factors determining the sensitivity of SARS-CoV-2 detection is the efficiency with which the designed primers and probes bind to target genes. Our results are supported by the study of Anantharajah A et al, which demonstrated differences between primers/probes recommended by the W.H.O. and the sensitivity of SARS-CoV-2 RNA detection. This team revealed that several nucleotide mismatches can contribute to false negatives although genetic diversity remains relatively low at the primer/probe binding sites [8].

As the specificity of the PCR step depends crucially on the primers, a simple defect can generate the absence or modification of the amplification. We also know that the mismatches that have been found at the level of the F and/or R primers can very likely induce the absence of amplification. Therefore, we were specifically interested in the last 5 nucleotides at the 3' end of the primers which seem to disturb the amplification more than the other mutated positions [22].

For real-time assays, the design of nonspecific primers can be minimized by selecting primers that have only one or two G/Cs in the last five nucleotides at the 3' end. This instability at their 3' ends makes them less likely to hybridize transiently and can cause non-specific amplification by DNA polymerase [22, 23].

Furthermore, the probes should not have sequence complementarity with the primers, and the TaqMan probes should not contain G at their 5' ends, this can quench the fluorescence of the reporter, even after cleavage [22]. As a result, the mutated positions at the level of the probes have rather an impact on the hybridization of the target on the side of the 5′ end. For this reason, we made a comparison between the seven RT-qPCR kits concerning the different mismatches located at the 3' end of the two primers F and R, at the 5' end of the probe, and the other positions for the Omicron SARS-CoV-2 variant. The results are detailed in Table 3.

First, we compared the seven kits according to the position of the mismatches at the Reverse primer. Indeed, we observed that there are more mutations outside the 3' end of the R primers than inside and this was found at the level of the seven RT-qPCR kits. However, some diagnostic kits still showed more mismatches at the last five nucleotides at the 3' end of the R primer than others. The number of mismatches varied between 17% and 35% with a total absence of mutations at this position in the Charite Germany kit (0%). This distribution showed a statistically significant difference between the seven RT-qPCR kits (*p*= 0.01) (Table 3).

Second, we compared the seven kits according to the position of the mismatches at the level of the Forward primer. Indeed, we have observed that there are sometimes more mutations outside the 3' end of the F primers and this was found in four RT-qPCR kits (Hong. Kong, NIH. Thailand, NIID Japan, and US CDC). However, sometimes the number of mutations was identical between those located at the 3' end and those outside this position and this has been demonstrated for the two kits Charite Germany and Pasteur Institute. To our surprise, the CDC China kit had more mismatches at the last five nucleotides than outside (55% vs. 45%, respectively). The mismatch distribution between the 7 kits at primer F level showed a significant difference (*p*=0.04) (Table 3).

Thirdly and lastly, we compared at the probe level this time the seven kits according to the position of the mismatches. Indeed, we have observed that there is no mutation at the 5' end of the probes marketed in the two kits Charite Germany and Pasteur Institute (00%). For the rest of the kits, we observed the presence of between 25% and 33% mismatch at the 5' end of the probes. Despite this almost identical distribution, we showed a statistically significant difference between the seven RT-qPCR kits (*p*=0.03) (Table 3).

**Table 3:** comparison between the seven RT-qPCR kits concerning the different mismatches located at the 3' end of the two primers F and R, at the 5' end of the probe and the other positions for the Omicron SARS-CoV-2 variant.

	**Reverse**		**Probe**		**Forward**	
**RT-qPCR kits**	**Other mismatches**	**3’ end mismatches**	**Other mismatches**	**5’ end mismatches**	**Other mismatches**	**3’ end mismatches**
**CDC. China**	15 (65%)	08 (35%)	14 (67%)	07 (33%)	05 (45%)	06 (55%)
**Charite Germany**	01 (100%)	00 (00%)	03 (100%)	00 (00%)	02 (50%)	02 (50%)
**HKU. Hong Kong**	15 (75%)	05 (25%)	15 (75%)	05 (25%)	18 (78%)	5 (22%)
**NIH. Thailand**	13 (68%)	06 (32%)	03 (75%)	01 (25%)	13 (65%)	07 (35%)
**NIID. Japan**	44 (65%)	24 (35%)	15 (75%)	05 (25%)	39 (64%)	22 (36%)
**CDC.US**	21 (78%)	06 (22%)	35 (73%)	13 (27%)	46 (100%)	13 (22%)
**Pasteur Institute**	05 (83%)	1 (17%)	00 (00%)	00 (00%)	02 (67%)	01 (33%)
***p*** **-value**	**0.01**		**0.03**		**0.04**	

Throughout the study, we based ourselves on the number of mismatches presented by the sequences of each RT-qPCR diagnostic kit compared to the 446 Algerian SARS-COV-2 sequences that we aligned. Finally, we cannot predict the effectiveness of a kit concerning this criterion alone. We agree that the presence of one or two mismatches between the 446 SARS-COV-2 sequences and the primers and/or probes is much more significant than the presence of 20 mismatches between 1 or 2 aligned sequences and the primers /probes. This is why we decided to compare this parameter which is the number of sequences carrying or not the mismatches between the seven RT-qPCR kits and the results are shown in Table 4.

We presented the number of Algerian SARS-CoV-2 sequences that presented or did not have significant mismatches in each type of sequence of the two primers and the probes (among the total of 446 already aligned for this study). We compared this number of SARS-CoV-2 sequences between the seven COVID-19 diagnostic kits already used in our study according to the presence (3' or 5' ends) or the absence of mismatches.

As mentioned in Table 4, we can conclude that there are two categories of diagnostic kits, there are those which show the presence of mismatches on a large part of the aligned Algerian SARS-CoV-2 sequences (i.e., the 446 sequences of departure), this concerns the two kits *NIH *Thailand (99.5%) and NIID Japan (99.5%). But some only show mismatches on a few or even no SARS-CoV-2 sequences. For this category, we noticed the almost total absence of mismatches between the 446 sequences and the sequences of the primers and probes of the four kits CDC China (99%), Charite Germany (100%), HKU Hong Kong (98%) and Pasteur Institute (99.5%). For the latest RT-qPCR kit, in particular the US CDC kit, we observed an almost total absence of mismatches between its primers/probes and the Algerian viral sequences only for the Reverse primer (98%). For the Forward primers and the probe, we rather found a presence of mismatches (99% and 96%, respectively) (Table 4).

Furthermore, we analyzed the distribution of these sequences according to the absence/ presence of mismatches between each COVID-19 diagnostic kit separately as illustrated in the *p* values line in Table 4. We analyzed the distribution of these sequences according to the absence/presence of mismatches between each COVID-19 diagnostic kit separately as shown in the p values line in Table 4. After the Bonferroni corrections, we have retained several interesting combinations that reflect our previous results. Indeed, we were able to demonstrate concerning the sequences of the Forward primers that the two kits NHD Thailand and NIID Japan proved to be less effective and specific to the Algerian version of the SARS-CoV-2 viral genome of the Omicron variant compared to the other kits (*p*=10^-6^). Concerning the Reverse primers and the fluorescent probes, we noticed a low efficiency for the three kits NHD Thailand, NIID Japan, and CDC US compared to the other kits (*p*=10^-6^).

**Table 4:** The 446 Algerian SARS-CoV-2 sequences comparison between the presence and the absence of mismatches in primers and probes for RT-qPCR kits.

**Diagnostic kits**	**Reverse primer**		**Probes**		**Forward primer**	
	**Absence of mismatches** n sequences (%)	**Presence of mismatches** n sequences (%)	**Presence of mismatches** n sequences (%)	**Presence of mismatches** n sequences (%)	**Presence of mismatches** n sequences (%)	**Presence of mismatches** n sequences (%)
**CDC China** ^a^	442 (99%)	004 (01%)	443 (99%)	003 (01%)	444 (99%)	002 (01%)
**Charite Germany** ^b^	446 (100%)	000 (00%)	446 (100%)	000 (00%)	438 (98%)	008 (02%)
**HKU. Hong Kong** ^c^	439 (98%)	007 (02%)	444 (99%)	002 (01%)	444 (99%)	002 (01%)
**NIH. Thailand** ^d^	001 (0.5%)	445 (99.5%)	001 (0.5%)	445 (99.5%)	001 (0.5%)	445 (99.5%)
**NIID Japan** ^e^	001 (0.5%)	445 (99.5%)	007 (02%)	439 (98%)	000 (00%)	446 (100%)
**CDC US** ^f^	440 (98%)	006 (02%)	016 (04%)	430 (96%)	002 (01%)	444 (99%)
**Pasteur Instituteg**	445 (99.5%)	001 (0.5%)	446 (100%)	000 (00%)	443 (99%)	003 (01%)
	a/d= **10**^-6^ b/d= **10**^-6^ c/d= **10**^-6^ f/d= **10**^-6^ g/d= **10**^-6^ a/e= **10**^-6^ b/e= **10**^-6^ c/e= **10**^-6^		a/d= **10**^-6^ b/d= **10**^-6^ c/d= **10**^-6^ f/d= **10**^-6^ g/d= **10**^-6^ a/e= **10**^-6^ b/e= **10**^-6^ c/e= **10**^-6^		a/d= **10**^-6^ b/d= **10**^-6^ c/d= **10**^-6^ f/d= **10**^-6^ g/d= **10**^-6^ a/e= **10**^-6^ b/e= **10**^-6^ c/e= **10**^-6^	
***p *** values*	f/e= **10**^-6^ g/e= **10**^-6^ a/b= NS a/c= NS a/f= NS a/g= NS b/c= NS b/f= NS b/g= NS c/f= NS c/g= NS f/g= NS		f/e= **10**^-6^ g/e= **10**^-6^ a/f= **10**^-6^ b/f= **10**^-6^ c/f= **10**^-6^ g/f= **10**^-6^ a/b= NS a/c= NS a/g= NS b/c= NS b/g= NS c/g= NS		f/e= **10**^-6^ g/e= **10**^-6^ a/f= **10**^-6^ b/f= **10**^-6^ c/f= **10**^-6^ g/f= **10**^-6^ a/b= NS a/c= NS a/g= NS b/c= NS b/g= NS c/g= NS	

N: number. *: Bonferroni correction (*p*<0.001), NS: No Significance

These results can be explained by the genetic component of the viral genome sequenced in Thailand, Japan, or even the United States. However, it is also evident as mentioned in the first Table that the kits designed in these countries have focused on the design of primers and probes on reduced regions. For example, the Thai and American kits designed their reagents only on the *N gene,* unlike other country's kits. The Japanese kit used a set of several target genes, but unfortunately, they targeted the S gene which is known for the frequent number of mutations and which is often not taken into consideration during sequencing.

It is interesting to know that the mismatches found in this study are either substitutions or deletions depending on the RT-qPCR kit used and depending on the target genes. We have encountered many substitutions whether about primers or probes. For example, for the Charite Germany kit, there is a substitution (T>C) at the 3' end of the F primer targeting the E gene and another substitution (G>A) at the 3' end of the primer F targeting the RdRP gene. For the deletions, we noticed the existence on one side of certain nucleotides absent on certain aligned Algerian SARS-CoV-2 sequences, and on the other side the absence of the total or partial sequence of the primers and/or probes on some or all of the aligned sequences. For example, the Chinese Hong Kong kit presents a deletion of the total sequence of the F primer targeting the *N *gene at the level of 7 Algerian SARS-CoV-2 sequences and also of the probe targeting the same gene at the level of 2 sequences aligned (Table 5).

**Table 5:** Mismatches type found in primers and probes for the Omicron variant in the 446 Algerian SARS-CoV-2 sequences compared with each kit target.

**Diagnostic kits**	**Target genes**	**Reverse 3’ end mismatches**	**Forward 3’ end mismatches**	**Probe 5’ end mismatches**
**CDC China Kit**	***N gene***	3 sequences with substitutions. 1 sequence with R primer deleted.	1 sequence with deletion.	1 sequence with substitution. 1 sequence with probe sequences deleted.
	***ORF1b gene***	1 sequence with substitution	/	1 sequence with substitutions.
**Charite Germany kit**	***E gene***	3 sequences with substitutions. 440 sequences with substitution in 5’end	/	/
	***RdRP gene***	/	5 sequences with substitutions.	/
**HKU. Hong Kong kit**	***N gene***	7 sequences with R primer deleted.	2 sequences with probe sequences deleted.	4 sequences with 3’end probe deleted.
	***ORF1b gene***	/	/	/
**NIH Thailand kit**	***N gene***	2 sequences with R primer deleted. 443 sequences with substitution.	443 sequences with 2 substitutions. 2 sequences with probe sequences deleted.	445 sequences with substitution.
	***N gene***	7 sequences with R primer deleted.	2 sequences with all F primer deleted.	5 sequences with 5’end probe deleted.
**NIID Japan kit**	***ORF1a gene***	R1: All sequences with 3 substitutions. R2: 439 sequences with substitutions. R3: 435 sequences with substitution.	F1: All sequences with substitution. F2: / F3: All sequences with substitution.	/
	***S gene***	/	F1: All sequences with substitutions. F2: All sequences with substitutions. F3: All sequences with substitutions.	/
**CDC US kit**	***N1 gene***	/	2 sequences with deletions in F primer.	16 sequences with probe sequences deleted. 414 sequences with substitutions.
	***N2 gene***	5 sequences with R primer deleted.	2 sequences with F primer deleted.	3 sequences with probe sequences deleted.
	***N3 gene***	1 sequence with 1 substitution.	1 sequence with F primer deleted. 444 sequences with substitution.	/
**Pasteur Institute kit**	***E gene***	/	3 sequences with 1 substitution.	/
	***RdRP IP2 gene***	1 sequence with substitution.	/	/
	***RdRP IP4 gene***	/	/	/

*: Mismatches found in the last five nucleotides of primer (3’end) or in the first five nucleotides of probe (5’end).

For ORF1a and S genes: only three F and R primers were used. No probes were used.

It has been proven that notable mismatches in the regions targeted by the primer/probe sets could affect the performance of RT-qPCR assays depending on their location and the nature of the substitution [24, 25]. The survey published on February 11, 2020, reported that N, E, and RdRp gene testing was quickly implemented by European laboratories according to *WHO* recommendations [26]. In addition, the team of Anantharajah et al highlighted a difference between the target genes used in the marketed kits. Indeed, it appears that tests targeting the N gene stand out from those targeting the E and RdRp genes for the detection of low-level viral loads [8]. This would imply that if the mutations affect the N genes, the detection tests targeting these genes become ineffective. So, in this study, we observed that the N genes targeted by several commercial kits underwent more mutations than the other genes as illustrated in Table 1. These observations confirm questions regarding the specificity of molecular tests for the diagnosis of COVID-19.

**Table 6:** Comparison of the frequency of mismatches found in this study with reference data (ECDC)

**Diagnostic kits**		**ECDC comparison**		**Reverse primer**		**Probe**	**Forward primer**	
**CDC China kit**		Reference		5.72		0.01	5.98	
		Our study		0.17	0.01		0.92	
**CDC US kit**		Reference		0.28		0.07	0.07	
		Our study		0.02	0.51		0.03	
**Charite Germany kit**		Reference		0.004		15.80	0.007	
		Our study		1	1		1	

Based on several observations, multiplexing tests with multiple target genes within a single PCR mixture could enable more reliable detection of SARS-CoV-2. We have already confirmed this in previous work on the detection of SARS-CoV-2 where we noticed that kits that target several genes at the same time gave more efficient results [27]. This could help future studies to design more specific and sensitive molecular tests.

Although ECDC does not provide direct genomic data, we can use their analyses and reports to interpret our analyses of SARS-CoV-2 mutations. Indeed, the mismatches observed in this study were compared with the Database of ECDC for all the diagnostic kits. We could make a comparison between our results and the Data shown on the ECDC website for CDC China, CDC US, and Charite Germany Kits. However, we could not compare with HKU Hong Kong, NIH Thailand, NIID JAPAN, and Institute Pasteur kits because some target genes used in this study were not found on the ECDC website.

All our comparisons have shown a different significant statistic between the results earned in this study compared to the Database ECDC. Moreover, only one mismatch frequency was found in our studies for the CDC China Kit, it shares the same frequency in the probe position for the ECDC Database. This observation does not influence the first conclusions since the diagnostic kit is a set of primers and probes, not just the probes.

Detection of the SARS-Cov-2 omicron variant by RT-qPCR was affected by the numerous mutations accumulated throughout the COVID-19 pandemic. Surveillance of the genomic sequence of SARS-CoV-2 in different regions of the world was strongly recommended by the WHO from the start of the pandemic. Early identification of mutations in critical regions of the SARS-CoV-2 genome is paramount to provide recommendations on specific diagnostic tests and ensure coverage of genetic variants circulating internationally and locally.

In this study, we found that the RT-qPCR kits CDC China, Charite Germany, NHD Hong Kong, and Pasteur Institute seem to be more effective and specific for the detection of the omicron viral genome of Algerian SARS-COV-2 and therefore for diagnosis of COVID-19 in Algeria. It would be interesting to study the effect of each nucleotide in destabilizing the hybridization of primers and probes with the Algerian SARS-CoV-2 sequences by making a correlation between the results of this study and the RT-qPCR test accomplished in Algeria. This perspective is currently being realized, we precisely designed primers that correctly identified the Omicron variant according to Algerian sequences. This prepares us for the emergence of future variants presenting new inconsistencies with the primers in the marketed kits. All the work in this direction (bioinformatics) demonstrates the importance of sequence monitoring, the need to predict the impact of mismatches, and the relevance of adapting molecular diagnostic tests to the evolution of pathogens. It is also important to emphasize that we have not carried out an "in vitro" comparison study between the different tests. Our conclusions are based solely on “in silico” bioinformatics analysis.
